# Stellate Ganglion Block for Long COVID Symptom Management: A Case Report

**DOI:** 10.7759/cureus.32295

**Published:** 2022-12-07

**Authors:** Mashfee H Khan, Kennedy P Kirkpatrick, Yi Deng, Krishna B Shah

**Affiliations:** 1 Anesthesiology, Baylor College of Medicine, Houston, USA; 2 Anesthesiology and Interventional Pain, Baylor College of Medicine, Houston, USA

**Keywords:** long-covid, stellate ganglion block, chronic fatigue, stellate ganglion, covid 19

## Abstract

Stellate ganglion block (SGB) is gaining increasing acceptance as a treatment modality for various medical conditions. It works by blocking neuronal transmissions which in turn alleviates sympathetically-driven disease processes. Many of the prolonged sequelae of long COVID are thought to be mediated by dysregulation of the autonomic nervous system, and SGB is being investigated as a potential option for symptomatic management of long COVID. This case report demonstrates the efficacy of SGB in a previously healthy patient for the management of long COVID symptoms including fatigue, post-exertional malaise, shortness of breath, and gastrointestinal symptoms.

## Introduction

COVID-19 (coronavirus disease 2019) resulted in substantial morbidity and mortality [1]. The long-term effects of the virus are still being extensively studied; however, patients who developed prolonged symptoms ranging from orthostatic intolerance, fatigue, shortness of breath (SOB), brain fog, anosmia, dysgeusia, and mental health complications, now termed “long COVID,” are being increasingly recognized [1]. Up to 30% of individuals infected with COVID-19 go on to develop long COVID with symptoms lasting indefinitely [1]. With the pathophysiology of long COVID not well understood, treatment regimens are diverse-ranging, investigative in nature, and have little support in current literature. 

Stellate ganglion block (SGB) is increasingly being used for treating various medical conditions. The overlapping features of these conditions, and the effectiveness of SGB in their symptom management, stem from blocking sympathetically mediated disease manifestations [2]. SGB blocks neuronal transmissions while reducing sympathetic activation and neurohormone levels. It has been effective in treating post-traumatic stress disorder, ventricular tachyarrhythmias, and hot flashes due to menopause, and is now being investigated as a plausible treatment for long COVID [2]. Symptoms of long COVID including anosmia and dysautonomia have shown improvement following SGB; however, this case report highlights the efficacy of SGB in alleviating long COVID symptoms limited to fatigue, post-exertional malaise (PEM), SOB, and gastrointestinal (GI) symptoms.  This manuscript adheres to the applicable EQUATOR guideline. Written HIPAA (Health Insurance Portability and Accountability Act) authorization for publication of this case report was obtained from the patient. Written consent was obtained from the patient to publish this article with a photograph of the patient.

## Case presentation

**Figure 1:**
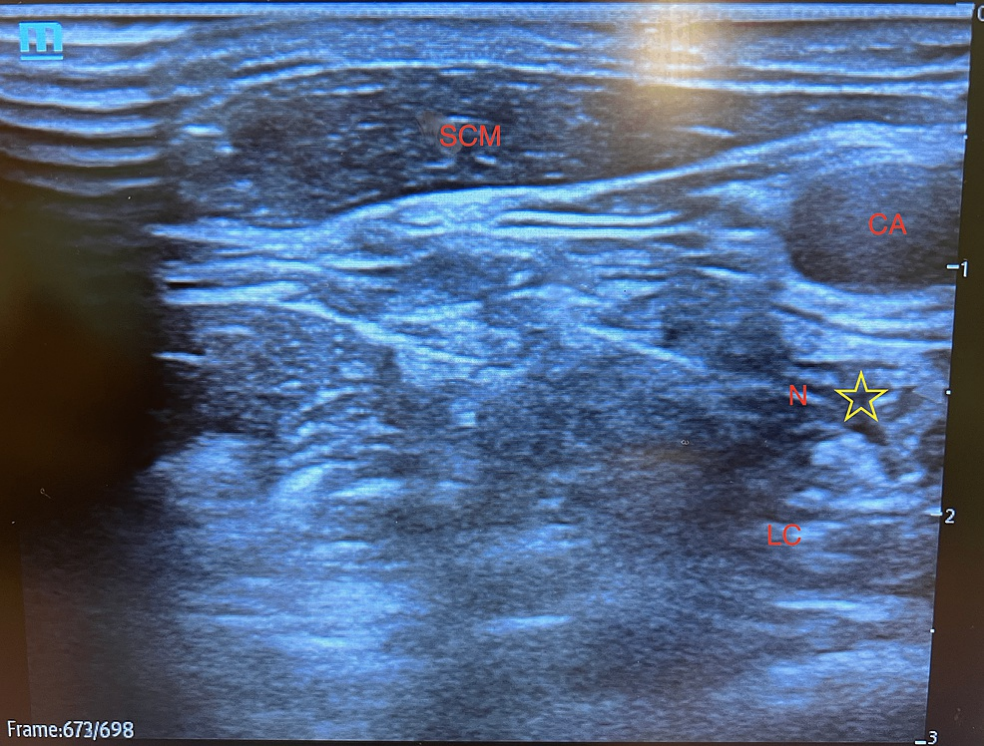
Ultrasound-guided stellate ganglion block at the level of C6 transverse process SCM: sternocleidomastoid muscle; CA: carotid artery; LCL longus colli muscle; N: needle; star symbol: sympathetic chain

**Figure 2:**
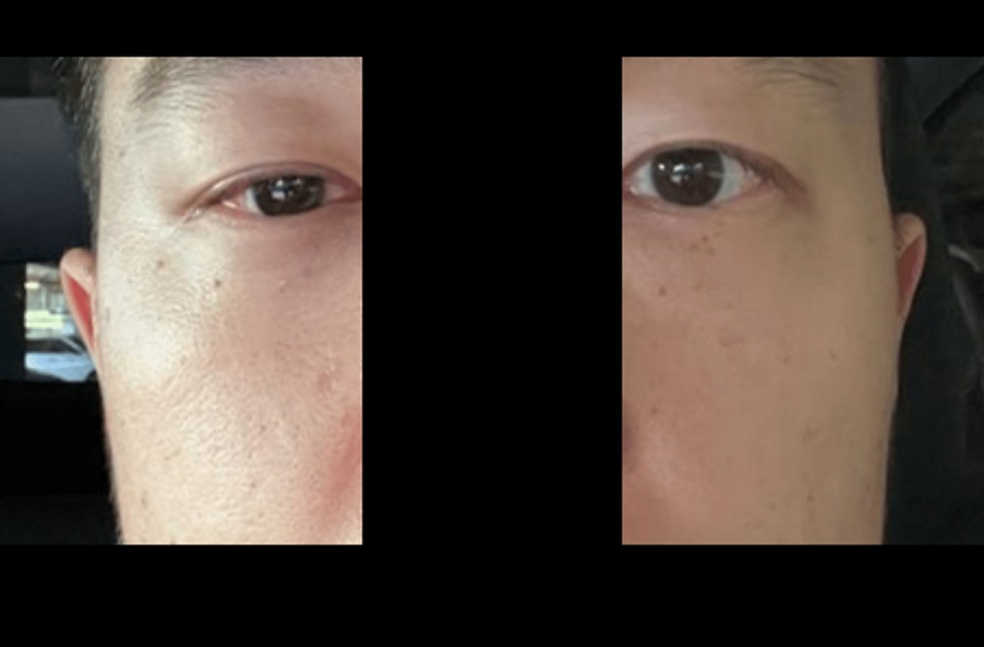
Confirmation of unilateral Horner's syndrome following stellate ganglion block.

A 38-year-old previously healthy man presented to the interventional pain clinic with a history of presumed COVID-19 from March 2020. The diagnosis was unable to be confirmed at the time due to a lack of COVID-specific testing early in the pandemic despite matching symptomatology and negative viral respiratory panel. After the acute phase of the infection, the patient described ongoing daily fatigue, SOB, PEM, persistent flu-like symptoms (sore throat, rhinorrhea, cough), myalgias, cold intolerance, and GI symptoms including diarrhea and intermittent abdominal pain for over two years. Extensive work-up for other etiologies was negative and a diagnosis of long COVID was made. Given the possible sympathetically mediated symptoms, a decision was made to proceed with a right-sided SGB under ultrasound guidance (Figure 1). The injectate contained 4ml of 0.25% (10mg) bupivacaine with 1ml (10mg) dexamethasone. He developed partial Horner’s syndrome (ptosis and chemosis of the R eye along with unilateral facial flushing) post-procedure as a confirmation of adequate blockade (Figure 2). His symptoms immediately improved the next day after the injection, with the most notable being fatigue and malaise. We then proceeded with a repeat SGB on the contralateral side with the same injectate solution 12 days later, with further improvement in SOB and GI symptoms. These improvements are described in Table 1. Standard ASA monitors were applied and checked every three minutes during both procedures.

**Table 1:** Long COVID symptom evaluation pre- and post-SGB *symptoms were rated on a 1-10 scale with 1 being the best and 10 being the worst; a questionnaire was administered to the patient before and after the stellate ganglion block. SGB: stellate ganglion block

	Pre-SGB	Post-SGB	Percent Change (Pre-SGB/Post-SGB)
General Symptoms			
Fatigue that interferes with daily life	8	4	50%
Symptoms that get worse after physical/mental effort	8	3	38%
Fever	0	0	-
Respiratory or heart symptoms			-
Shortness of breath	5	2	40%
Cough	3	1	33%
Chest pain	0	0	-
Palpitations	0	0	-
Neurologic symptoms			
Difficulty concentrating; "brain fog"	0	0	-
Headache	0	0	-
Sleep disturbance	3	2	67%
Dizziness upon standing	0	0	-
Pins-and-needles feelings	2	1	50%
Change in smell or taste	0	0	-
Depression or anxiety	0	0	-
Digestive symptoms			-
Diarrhea	2	1	50%
Stomach pain	2	0	0%
Other			-
Joint or muscle pain	4	1	25%
Rash	2	1	50%

## Discussion

SGB has been used effectively in the treatment of long COVID-associated anosmia and dysautonomia in literature to date. This case report showcases the potential effectiveness in the treatment of other prominent long COVID symptoms including fatigue, PEM, SOB, and GI disturbances.  

Long COVID dysautonomia may be driven by hyperinflammation-induced sympathetic overdrive [3]. Existing treatment modalities for this are sparse, but the utilization of SGB in a select group of patients has been shown to reduce or eliminate symptom severity [3,4]. The autonomic nervous system and inflammatory system are closely intertwined with a bidirectional communication cascade that allows for both rapid response to infection and negative feedback to prevent hyperinflammation [5]. Long COVID dysautonomia seems to result from the dysregulation of this relationship [5]. In acute tissue injury caused by COVID-19, vasomotor dysfunction and neurogenic inflammation can occur causing dysregulations in neuronal plasticity [4,6]. This results in chronic sympathetic hyperresponsiveness, which makes SGB a reasonable treatment modality to directly target the sympathetic system. SGB may treat dysautonomia by increasing both cerebral and regional blood flow, in turn, resetting the autonomic nervous system function [4]. Further research is needed to elucidate its exact mechanism; however, with the lack of other treatment options, symptom management with SGB has shown promising results in select case reports to date [3,4]. 

Anosmia and dysgeusia are two common sequelae of COVID-19 infections that can persist for up to 12 months from the onset of symptoms [7,8]. Although research is limited, SGB has been shown to reduce or eliminate anosmia in select groups of patients with long COVID [8,9]. The underlying mechanism is postulated to be the upregulation of cerebral blood flow (CBF), either to olfactory and gustatory centers of the brain or to peripheral chemoreceptors [7-9]. However, with the SGB effects lasting longer than that of local anesthetics, alternative mechanisms are also likely at play. Further investigation is needed to elucidate the benefits of SGB in long COVID-induced anosmia and dysgeusia. 

The amount of local anesthetic used in SGB has not been standardized, with one study showing 4ml of injectate in an ultrasound-guided SGB resulting in successful Horner’s syndrome in 100% of patients while another study showing 2ml of injectate was successful in causing spread below the C7-T1 junction in 100% of patients [10]. A previous case report for SGB performed for long-COVID used 4ml of 0.25% bupivacaine [9]. Dexamethasone was added to the local anesthetic to prolong the block effect as reported in previous literature [11]. 

Typically, bilateral SGBs are not done simultaneously due to concern for either airway compromise from bilateral recurrent laryngeal nerve palsy or respiratory impairment from bilateral phrenic nerve palsy [11]. There are rare reports of bilateral SGBs being performed, for instance in a patient that was intubated and mechanically ventilated [12]. In case reports thus far, SGB for long COVID is done unilaterally and then repeated on the contralateral side. However, the timing between SGBs is not well defined. Our patient underwent contralateral SGB 12 days after the initial injection. In prior case reports, the time between blocks has ranged between 24 and 72 hours [4,9]. Among patients receiving SGB for long COVID, all to date have reported further improvement after administration of the contralateral block [4,9]. Our patient also endorsed further improved SOB and GI disturbances after the contralateral injection. To date, no other case report has attempted repetitive unilateral SGB in the management of long COVID symptoms. Our patient did not elect to repeat SGB after the initial series due to marked symptom improvement. However, in patients with prolonged or recurring symptoms, it may be warranted to repeat the blocks due to their relatively low-risk profile. Further research is needed to address the validity and timing of repeated blocks in this setting. One limitation of this study is that this case was not a laboratory-confirmed case of COVID-19, but rather a clinical diagnosis due to the unavailability of COVID-specific testing at the time of diagnosis.

Neurolysis of the stellate ganglion is another modality that can be considered for refractory symptomatic long COVID. It is rarely used due to the risk of permanent Horner syndrome and motor paralysis; however, case reports have demonstrated its efficacy in the treatment of pain for various oncologic conditions as well as refractory ventricular fibrillation [13,14]. Given the risk of complications with neurolysis, this procedure should be discussed in detail with patients to weigh its potential risks and benefits.  

## Conclusions

The use of SGB as a treatment modality for many sympathetically driven disease processes is increasing. SGB may treat dysautonomia by increasing both cerebral and regional blood flow, in turn, resetting the autonomic nervous system function. Its beneficial effects on prolonged and often debilitating symptoms associated with long COVID are worth further exploration. Currently, the evidence for using SGB to alleviate symptoms associated with long COVID is anecdotal and limited to a few case reports. Further research is needed to elucidate the optimal dosing of injectate as well as the validity of repeated blocks.
